# Copper Metabolism of Newborns Is Adapted to Milk Ceruloplasmin as a Nutritive Source of Copper: Overview of the Current Data

**DOI:** 10.3390/nu10111591

**Published:** 2018-10-30

**Authors:** Ludmila V. Puchkova, Polina S. Babich, Yulia A. Zatulovskaia, Ekaterina Y. Ilyechova, Francesca Di Sole

**Affiliations:** 1 Laboratory of Trace Elements Metabolism, ITMO University, Kronverksky av., 49, 197101 St.-Petersburg, Russia; ikaterina2705@yandex.ru; 2 Department of Molecular Genetics, Research Institute of Experimental Medicine, Acad. Pavlov str., 12, 197376 St.-Petersburg, Russia; 3 Department of Biophysics, Peter the Great St. Petersburg Polytechnic University, Politekhnicheskaya str., 29, 195251 St.-Petersburg, Russia; 4 Department of Zoology, Herzen State Pedagogical University of Russia, Kazanskaya str., 6, 191186 St.-Petersburg, Russia; babich.polina@gmail.com; 5 Department of Neurosurgery, Stanford University School of Medicine, Stanford, CA 94305, USA; yuliaz@stanford.edu; 6 Department of Physiology and Pharmacology, Des Moines University, Des Moines, IA 50312, USA; francesca.disole@dmu.edu

**Keywords:** embryonic type copper metabolism, milk ceruloplasmin, baby formula

## Abstract

Copper, which can potentially be a highly toxic agent, is an essential nutrient due to its role as a cofactor for cuproenzymes and its participation in signaling pathways. In mammals, the liver is a central organ that controls copper turnover throughout the body, including copper absorption, distribution, and excretion. In ontogenesis, there are two types of copper metabolism, embryonic and adult, which maintain the balance of copper in each of these periods of life, respectively. In the liver cells, these types of metabolism are characterized by the specific expression patterns and activity levels of the genes encoding ceruloplasmin, which is the main extracellular ferroxidase and copper transporter, and the proteins mediating ceruloplasmin metalation. In newborns, the molecular genetic mechanisms responsible for copper homeostasis and the ontogenetic switch from embryonic to adult copper metabolism are highly adapted to milk ceruloplasmin as a dietary source of copper. In the mammary gland cells, the level of ceruloplasmin gene expression and the alternative splicing of its pre-mRNA govern the amount of ceruloplasmin in the milk, and thus, the amount of copper absorbed by a newborn is controlled. In newborns, the absorption, distribution, and accumulation of copper are adapted to milk ceruloplasmin. If newborns are not breast-fed in the early stages of postnatal development, they do not have this natural control ensuring alimentary copper balance in the body. Although there is still much to be learned about the neonatal consequences of having an imbalance of copper in the mother/newborn system, the time to pay attention to this problem has arrived because the neonatal misbalance of copper may provoke the development of copper-related disorders.

## 1. Introduction

A suboptimal content of micronutrients in a mother’s diet during pregnancy and lactation may be a cause of developmental defects in newborns. The data show that severe neonatal defects can be prevented by correcting a mother’s diet (e.g., through iodine, folate, and iron supplementation) [1,2,3,4,5,6,7,8]. Fewer clear effects of a deficiency/excess of copper, one of the most important micronutrients, have been reported in fetuses/neonates [9]. In mammals, about two dozen enzymes, which control the basic cellular processes—respiration, antioxidant defense, the formation of connective tissue, neurotransmitter synthesis, neuropeptide processing, iron transport, and others—require copper as a cofactor for their activities [10,11]. Moreover, copper operates as a secondary messenger in some signaling pathways [12]. In adult mammals, a copper deficiency or the excess of copper from dietary factors is rarely observed. The exceptions are the cases of copper deficiency in agricultural animals in geochemical provinces with cobalt and/or selenium and/or copper or in areas with soil that is naturally high in molybdenum [13,14]. However, an imbalance of copper can also be developed from the surgical removal of the small intestine, genetic disease, and diseases that alter micronutrient metabolism [15,16]. The first clinical sign of a copper deficiency is anemia due to the disturbance of iron metabolism [17]. The inherited defects in copper transport cause the development of both copper deficiency and accumulation simultaneously because the loss of copper from transporting proteins results in copper accumulation in inappropriate cellular compartments and the deficiency of bioavailable copper. As a result, copper dyshomeostasis develops [16,18]. The liver and the brain, the organs with the most intensive copper metabolism [19], are affected first by the defects of copper transport. In these organs, signs of toxic copper accumulation and cuproenzyme deficiency manifest earlier than in other organs.

In newborns, the mechanisms for excreting copper through bile and controlling copper absorption in the small intestine do not operate [20]. Therefore, dietary factors may cause copper misbalance in the early stages of postnatal development. Copper deficiency in the food of pregnant and nursing females causes a lack of cuproenzyme activity, multiple developmental aberrations, teratoma formation, and death among fetuses or offspring in the early postnatal stages [21,22]. In babies fed cow milk (which has a much lower concentration of copper than does human milk), copper-dependent anemia almost inevitably develops [23]. At the same time, even a small excess of copper in tap water can be fatal to infants with inborn defects of copper transport in cells (childhood cirrhosis associated with copper [24,25] and cirrhosis of Indian childhood [26]). The aims of the present review are to emphasize the dependence of copper homeostasis in newborns on food sources and to pay attention to the possible long-term consequences of copper imbalances in neonates.

## 2. Biological Roles of Copper

Copper is an essential component of the cells of almost all modern organisms. It was involved in the metabolic processes in the early stages of the Earth’s evolution, possibly at the beginning of the Great Oxygenation Event, when it became bioavailable due to the oxidation of insoluble sulfides [27]. In aqueous media, copper ions have two stable oxidation states Cu(I)↔Cu(II), and both forms can have high toxicity [28]. Because copper ions have strong coordination properties, they can readily form coordination compounds with ligands that contain carboxyl, amine, pyrrole (imidazole, indole), pyridine, hydroxyl, nitrile, and, especially, thiol donor groups. Depending on the chemical nature of the ligands and the geometry of the coordination sphere, copper displays a wide range of redox potentials. A combination of these properties makes copper a valuable cofactor for enzymes catalyzing redox reactions. Cuproenzymes catalyze different redox reactions in which copper is cycled in Cu(I)↔Cu(II) states and serve both as an electron donor and an electron acceptor depending on the stage of the catalytic cycle. The most abundant cuproenzymes catalyze the electron transfer to dioxygen. In the active centers of cuproenzymes, copper is typically tightly bound by 4, 5, or 6 ligand groups and cannot be easily released or removed from the enzyme under physiological conditions [28]. The largest fraction of copper that is present in mammals is contained in the active centers of cuproenzymes and does not possess toxic properties. The physiological yrole of major mammalian cuproenzymes—their structure, function, and localization in the cell and in the organism—and their gene expression has been actively studied for many decades. The established data on these cuproenzymes are briefly summarized in Table 1.

The native tertiary structure of cuproenzymes is formed during the successful metabolic insertion of copper ion(s). The removal of copper from holoenzymes in vitro or defects in the processes of the metalation of apo-enzymes in vivo can cause the disruption of the tertiary structure and the loss of catalytic functions [41]. Therefore, copper is considered to be both a catalytic and a structural cofactor of cuproenzymes.

In the past few years, evidence of the existence of a regulatory role of copper has accumulated. This role has remained elusive for a long time, although the first evidence for the involvement of copper in the regulation of endothelial cell growth was obtained almost 40 years ago [42]. Recently, it was shown that intracellular and local extracellular changes in copper concentration influence the activity of transcription factors NF-κB, nuclear factor kappa-light-chain-enhancer of activated B cells [43], and HIF1, hypoxia-inducible factor 1 [44]. These factors regulate the expression of several dozens of genes, including genes whose products take part in the reprogramming of energy metabolism in tumor cells. X-linked inhibitor of apoptosis protein (XIAP), which is a common member of several signaling pathways, can bind copper. This binding leads to the release of caspase 3, which triggers XIAP-mediated apoptosis [45,46]. Copper performs a number of important functions, including the following: taking part in the Ras/MAP-kinase (Ras/Mitogen-activated protein kinase) [47] and Raf of murine oncogene sarcoma homologue B1-dependent [48] signaling pathways; modulating the function of growth factor receptors [49,50], γ-aminobutyric acid [51], and glutamate receptors [52]; regulating cyclic-AMP-dependent lipolysis [53]; and inducing Golgi-complex independent secretion of interleukins and cytokines [54]. It has also been shown that copper controls organogenesis and cell differentiation in embryo development [55].

Two prerequisites are implied from the existence of copper regulatory functions: (1) a pool, in which copper is accumulated and from which it is liberated on demand, and (2) copper-regulated sensors. In cells, two copper pools that may take part in the rapid, local change of copper concentrations exist (Table 2).

The first pool is a system that includes metallothionein-Cu(I), glutathione (Cu(I)/Cu(II)), and COMMD1-Cu(II) (Copper Metabolism gene MURR Domain 1), which possibly controls the changes of the copper concentration in the cytosol [69]. The members of this system can bind copper, change its oxidation state, and share it on demand with the cuproenzyme metalation, signaling pathways, and excretion routes. The second pool is associated with the mitochondria, which play an important role in copper homeodynamics and may be viewed as an intracellular copper depot [70]. It has been shown that in yeast and mammals, the mitochondria accumulate copper from the cytosol and release it back with the help of a copper ligand, which is a low molecular weight substance (~1 kDa) whose structure has not yet been identified. Therefore, there are means for rapid changes in the copper concentration in the cytosol. The existence of a kinetically labile copper pool, which is predominantly localized in the mitochondria and the Golgi apparatus, was shown by synchrotron X-ray fluorescence microscopy [71]. Local changes in the extracellular space near the cell membrane may occur when copper is secreted in complexes with interleukins, cytokines, growth factors, and metallothioneins under stress conditions. Copper-regulated sensors have been characterized in yeast, insects, and mammals (Table 2). In mammals, they include lysyl-oxidase-like proteins (LOXL1-4), and an imbalance in LOXL levels promotes tumor and metastasis growth. Another example is specificity protein 1 (Sp1), which is a transcription factor regulating the activity of the copper transporter 1 (CTR1) gene (see Table 3). Studies of copper’s regulatory role are relatively novel, and to date, there have been insufficient data to examine the consequences of impaired copper-dependent gene regulation.

## 3. Transport of Copper to the Places of Cuproenzyme Formation in Adult Mammals

The formation of holo-cuproenzymes is dependent on extracellular and intracellular copper traffic. Extracellular transport involves copper absorption from digested food in the gastrointestinal tract lumen and its transfer into the bloodstream, by which copper is delivered to the liver. Food copper can be in both oxidation states, Cu(I) and Cu(II). The physiological role of enterocytes is to absorb copper from the gastrointestinal tract and put it into circulation. Enterocytes contain two proteins that can import copper into cells. The first is transmembrane protein CTR1, a highly affine copper transporter 1. It is a transmembrane homotrimer, each subunit of which contains 3 α-helices, forming a transmembrane domain of 9 α-helices. The N-terminal extracellular domain contains 3 sites for Cu(II) or Cu(I) binding. The short C-terminal domain contains a copper-binding His-Cys-His-motif for Cu(I) [72]. The CTR1 gene is expressed in all cells. However, in polarized Caco2 cells and T84 cells, models for intestinal crypt cells, and in Madin Darby canine kidney cells, CTR1 is localized at the basolateral membrane, and it has been demonstrated that ^64^Cu is taken up through the basolateral membrane [85]. Therefore, basolateral hCTR1 imports copper from the blood to the intracellular cuproenzymes of the enterocytes.

The second copper importer is a divalent metal transporter 1 (DMT1), a member of the proton-coupled metal ion transporter family, which mediates the transportation of ferrous iron from the lumen of the intestine into the enterocyte. It consists of the only subunit with 12 α-helices, which form a transmembrane domain; both of its polypeptide terminals are oriented into the cytosol [82]. It is localized at the apical side of the intestine and plays a relevant role in physiological Cu(I)/Cu(II) entry [83,86]. Mice lacking intestinal DMT1 absorbed ^64^Cu from an intragastric dose to the same extent as did DMT1(+/+) mice [87]. Moreover, the mice with intestinal epithelial cell-specific Ctr1 knockout exhibited striking neonatal defects in copper accumulation in the peripheral tissues, hepatic iron overload, cardiac hypertrophy, and severe growth and viability defects. One round of postnatal administration of copper sulfate solution eliminated these defects [87,88]. These data suggest the significance of a critical neonatal metabolic requirement for copper that is provided by intestinal Ctr1. These studies in vivo identify Ctr1 as the major factor driving intestinal copper absorption in mammals. CTR1 imports copper as Cu(I). In mammals, two ferric reductases were found. One of them, duodenal cytochrome b (Dcytb), belongs to cytochrome *b_561_* [89]. The second are the proteins of the six-transmembrane epithelial antigen of the prostate (STEAP) metalloreductase family [90,91]. In an enterocyte, both can reduce Cu (II) to Cu(I). In the bloodstream, the absorbed copper is transferred by albumin or α-2-macroglobulin or Cu(His)_2_ in the oxidation state of Cu(II) [92].

The intracellular pathway includes copper delivery to the places where apo-cuproenzyme metalation occurs (i.e., the cytosol, the mitochondria, and the lumen of the Golgi complex). Inside the cells, the problem of safe copper transport is solved by a system of transporter proteins, which bind copper in a Cu(I) state [91]. In general, this system has been preserved in the evolution of eukaryotes. In mammals, the copper transport system (CTS) contains the largest number of components, and their patterns and expression levels are specific to the tissues and stages of ontogenesis. These proteins share a common trait, which is a copper-binding domain that typically contains a motif with two cysteine residues (Cys-X-Cys or Cys-X-X-Cys, where X is any amino acid). The domain is capable of bidentate Cu(I) coordination. The total length of the domain with the cysteine motif is comprised of dozens of amino acid residues. Their composition and sequence tune the affinity of the protein to copper and its abilities to accept or deliver copper ions. The proteins, which are also known as Cu(I)-chaperones, form transport chains and pass copper to each other via direct protein–protein contact, cycling between the holo-form and the apo-form. The direction of the transportation is determined by the increasing affinity to copper ions along the chain, which provides for the delivery of copper from the extracellular space to various cell compartments. Transporters have two domains for interacting with their respective partners. One domain is characteristic of the apo-form and facilitates binding to the copper donors, and the other domain enables the recognition of a copper recipient in its apo-form. Cu(I)-chaperones that insert copper into the active centers of cuproenzymes have domains for interacting with the apo-forms of these enzymes. While all the transporter proteins share the same principles and mechanisms of copper transfer, they can be naturally classified into soluble and integral transmembrane proteins (pore-like transporters or active pumps).

Copper is transported into the cell by the CTR1 protein, which is a universal high affinity copper importer (Table 3). The transport does not require ATP, and this protein has a highly selective Cu(I) pore [72]. The knockout of the *Ctr1^−/−^* gene in mice is lethal, and the embryos die in the first half of gestation, as well as display globally impaired morphogenesis [72]. The extracellular copper donors for mammalian CTR1 can be ceruloplasmin [93,94], albumin, and α-2-macroglobulin [95]. Cu(I), which crosses the membrane through the CTR1 pore, is bound by the cytosolic domain of this protein [96]. Then, the ion is passed to the cytosolic chaperones (CCS, COX17, ATOX1) that deliver copper to SOD1, mitochondria, and Cu(I)/Cu(II)-ATPases, respectively [76,77,78,79]. In mammals, there are two P1-type copper transporting ATPases: ATP7A (Menkes ATPase) and ATP7B (Wilson ATPase). These proteins were named, respectively, for the hereditary diseases (Menkes disease and Wilson disease) that are associated with the loss of the respective functions of each protein. The translocation of copper from the cytosol to the lumen of the Golgi complex is ATP-dependent and coupled with copper oxidation to Cu(II) [81].

The low-affinity copper transporter 2 (CTR2 protein) is homologous to CTR1 through its primary structure and channel-forming domain architecture, and it is localized to the membranes of endosomes and lysosomes [74,75]. The proteins of the STEAP metalloreductase family are also present in those locations, including cupric reductase STEAP4, which reduces Cu(II) to Cu(I) in endosomes [97]. Therefore, STEAP4/CTR2 activity may recycle copper from cuproenzymes that gets into the endolysosomal space during endocytosis or macroautophagy and return the copper to a bioavailable pool. Perhaps CTR2 controls the entry of copper into cells because copper is accumulated in *Ctr2*^−/−^ strains [75].

Copper is not accumulated by the proteins of the transport system, and these proteins may be viewed as a temporary package for safe delivery. Therefore, the physiological role of the CTS comprises copper importation, metalation of cuproenzymes, copper recycling, and copper excretion from cells. Because of the CTS, there are no ‘free’ copper ions in a cell [98]. However, copper disbalance can result in the appearance of ‘free’ copper and the generation of reactive oxygen species (ROS) and oxidative stress, as well as a decrease in bioavailable copper leading to cuproenzyme deficiency [99]. As a result, neurodegeneration, oncological, and cardiovascular disorders develop [18]. The increase of ‘free’ copper levels also poses a risk of zinc–copper displacement in zinc-finger transcription factors, which may cause global changes in the regulation of gene expression [100]. ‘Free’ copper ions were also shown to be able to disrupt active sites of [Fe–S]-metalloproteins [101], which control electron transport, DNA synthesis and repair, the regulation of gene expression, iron metabolism, and so on [102,103,104].

## 4. Copper Turnover in the Body of Adult Mammals

In the extracellular fluids of multicellular organisms, such as in the intracellular space, copper is coordinated by various carriers. Copper is found in ceruloplasmin (Cp), albumin, α-2-macroglobulin, and the bis-histidine complex [95,105,106,107]. In humans, more than 95% of copper is bound to Cp [105]. In mice, ceruloplasmin includes only about 60% of serum copper, and about 40% of copper is associated with α-2-macroglobulin [92]. Experiments with radioactive copper have shown that copper is absorbed in the intestine and passed through the intestinal cells to the bloodstream, where it is bound by albumin and transported to the liver. Near the plasma membranes of hepatocytes, copper from albumin is converted to His_2_Cu(II) and hepatocytes absorb copper from the latter complex. Inside these cells, copper is inserted in Cp and intracellular cuproenzymes, bound by metallothionein, or excreted in bile [108,109,110]. It is possible that the copper, which was inserted in Cp, is secreted back into the bloodstream and distributed to the organs [94]. Cp is an N-glycoprotein that binds 6–9 copper atoms, and six of them are tightly bound in active centers, while the others are weakly associated with the peptide chain [111]. In ceruloplasmin, the labile copper atoms can be replaced by zinc atoms [112]. Cp has many functions, and it is classified as a “moonlighting” protein [36]. It belongs to the family of blue multicopper (ferr)oxidases [37], and the major function of Cp is to facilitate iron redox transitions, which are required for transferrin/transferrin receptor-mediated iron transport through membranes. In vivo Cp oxidizes dopamine, serotonin, epinephrine, and norepinephrine and thus inactivates these hormones. Cp is an acute phase protein. Its level increases several times in inflammation, ovulation, pregnancy, lactation, and so on. Cp demonstrates weak antioxidant activity towards ROS. Cp is possibly the strongest regulator of neutrophil oxidative status and apoptosis [113]. Cp can also serve as a copper donor for non-hepatocyte cells [94]. In pulse-chase experiments, it has been shown that copper atoms associated with Cp are transferred into the cytosol of non-hepatic cells following the binding of Cp by a protein of the cell membrane [109]. Candidate proteins for Cp binding include the Cp receptor [114,115,116], CTR1 [93], or STEAP2 [94]. It was shown that in non-hepatic cells [^64^Cu]Cp and [^125^I]-Cp pass on [^64^Cu] into the cytosol, and the protein moiety (apo-Cp) is absorbed by endocytosis, desyalated in endolysosomal vesicles, and then returned into circulation. This Cp with a processed carbohydrate moiety is then captured by endocytosis in hepatocytes through the group-specific receptor of acidic desalted glycoproteins [117,118]. Cp, containing copper atoms that do not dissociate at low pH, is secreted into bile [119]. Thus, in adult mammals, Cp plays important role in supporting copper balance outside the cells.

## 5. Ontogenetic Changes in Copper Metabolism in Mammals

The ontogenetic development of mammals relies on two systems of cuproenzyme metalation, which successively operate in the liver. The first system corresponds to the embryonic type of copper metabolism (ETCM). It is active in prenatal and early postnatal stages of development, and then it is substituted by the adult type of copper metabolism (ATCM). Note that these abbreviations are not widely used; we propose them here for readability. The phenotypic traits of ETCM include the absence of regulated copper absorption in the small intestine (copper freely passes through the wall of the intestine), low copper and Cp levels in the blood serum (3–4 times lower compared with those of adults), the absence of copper excretion through bile when copper is excreted with urine, and copper accumulation in the liver [19,20,120,121,122]. The concentration of metallothionein-associated copper in the blood serum of rat newborns is 2 times higher than that in adult rats [121]. The distribution of copper between the liver and the blood in ETCM and the pathways of its excretion correspond to those observed in Wilson’s disease [16].

In ETCM, the blood serum copper status correlates with hepatic copper metabolism [120,121]. Therefore, hepatic Cp-gene expression is low; the expression of *Atp7b* is practically absent. The translocation of copper to the Golgi complex is implemented only by ATP7A; consequently, Cp is metalated by ATP7A but not by ATP7B. The activity of the *Sod1* gene and the level of holo-SOD1 are decreased by 30% compared with those of adults. However, the activity of the *Ccs* gene is decreased by a factor of 10 [121]. It may be suggested that holo-SOD1 formation in newborns is performed with the help of the MT/glutathione pair in the cytosol or in the intermembrane space of the mitochondria [69,70]. Despite the rapid, almost exponential, accumulation of copper in the liver cells, the activity of the *Ctr1* gene coding for the major universal copper importer is low, and its expression level is ~10% of that in adults. The low expression level of *Ctr1* in the liver of newborns is accompanied by high expression levels of *Ctr2* [121]. It is possible that CTR1 is not the main route of copper importation into the liver during this stage of development, which is characterized by ETCM. During the transition from ETCM to ATCM, the copper concentration in the liver drops abruptly, while the serum concentrations of Cp and Cp-associated copper increase. The change in the type of copper metabolism accompanies profound changes in the expression pattern of copper transporters in the liver: the *Atp7a* gene is repressed, and the *Mt* gene is downregulated, while the *Sp1*, *Atp7b*, *Cp*, *Ctr1, Atox1*, and *Ccs* genes are activated [120,121,122,123,124]. In the intestines, the transition from ETCM to ATCM occurs through the increased abundance and altered localization of Ctr1, Atp7A, and Atp7B [125], and as a result, the mechanism controlling copper absorption is formed [126].

In adult mammals, the brain, similar to the liver, is an organ with high copper concentrations [19]. This fact is related to the high concentrations of cuproenzymes in the brain cells. In addition to ubiquitous cuproenzymes (COX and SOD1), the brain contains copper-dependent enzymes that take part in iron transport, the processing of the pro-neuropeptides, the metabolism of neurotransmitters, and the synthesis of melanin (Table 1). The distribution of the enzymes and the respective copper content in brain tissue are not uniform [19,127,128]. The primary stages of copper delivery to the brain are unknown, and the specific ontogenetic features are not known either. During development, the concentration of copper in the brain does not change dramatically (in contrast with the liver), but it significantly increases in some regions (the cortex, hippocampus, and cerebellum) [128], possibly in accordance with the synthesis of brain-specific cuproenzymes during differentiation, as shown on the PC12 cells [129]. Also, the differentiation of PC12 cells into neurons induces metallothionein-3 expression, thereby resulting in intracellular copper accumulation [130]. The cerebrospinal fluid contains copper and Cp, and their concentrations are approximately 100 times lower compared with the blood serum. Moreover, they do not change during ontogenetic development [131]. Therefore, in the mammalian brain, intimate changes occur in the copper metabolism during the transition from ETCM to ATCM, which have not yet been evaluated.

In many regions of the brain, the synthesis of Cp splice-isoforms (secretory Cp and Cp associated with the membrane through glycophosphatidylinositol anchor, GPI-Cp), which are formed by the alternative splicing of the primary transcript, occurs [132]. GPI-Cp is required for iron efflux from cells in the central nervous system [38]. The synthesis of secretory Cp is stimulated by the adjacent endothelial cells, which form the blood–brain barrier [133] through the synthesis of interleukin-6 activating transcription of the *Cp* gene [134]. In some brain regions, secretory Cp is used as a ferroxidase [135]. In vivo, the formation of Cp splice forms is shifted towards GPI-Cp during neuronal differentiation [128]. Generally, the brain copper metabolism is unrelated to the changes that occur in the liver during the early stages of development. However, the facts are enough to consider that ontogenetic changes in the liver and brain are controlled by transcriptional and posttranscriptional regulation of the genes for extracellular and intracellular copper transporters.

## 6. Copper Metabolism in the Mammary Gland through Milk Ceruloplasmin Production

In adult mammals, copper assimilated in the small intestine is typically absorbed completely by the liver in several minutes. In approximately 90 min, copper is returned to the bloodstream as a component of serum Cp [136,137]. However, in lactating rats and humans, ~30% of assimilated copper (the total amount of which is 9–10 times higher compared with non-pregnant animals) bypasses the liver and is absorbed by the cells of the mammary gland [138,139]. In 30 min, this copper can be found in the milk [138]. The dynamics of copper transfer in milk perfectly coincide with the dynamics of the secretion of [^14^C]Cp into the milk [140]. This has been shown in pulse-chase experiments, in which a rat (on the fifth day of lactation) was injected intraperitoneally with labeled amino acids; at different time intervals, samples of blood were collected from a catheter in the neck vein, and milk was obtained by manual milking. In the samples, Cp was precipitated by antibodies to rat ceruloplasmin, and [^14^C]-Cp was measured. The mRNA coding the secretory form of Cp is found in the transcriptome of the cells of the lactating mammary glands. The length of milk Cp-mRNA and the molecular mass of milk Cp do not differ from hepatic Cp-mRNA and plasma Cp correspondingly [140,141]. Milk Cp possesses oxidase and ferroxidase activities [142,143,144]. In humans, the structure of glycan chains in milk Cp is different from serum Cp, as indicated by two dimensional immunoelectrophoresis with lectins [144]. It is likely that the milk Cp glycan moiety has no N-acetyl neuraminic acid residues. The concentration of milk Cp is the highest in the colostrum and decreases during lactation [142,143,144].

In the mammary gland cells, strong and rapid upregulation of the *Cp* gene, as well as the *Ctr1* and *Atp7b* genes, which provide Cp metalation, are observed shortly before the end of gestation [145]. During lactation, the expression levels of the *Cp*, *Ctr1*, and *Atp7b* genes gradually decrease according to the change in the Cp concentration in the milk. The same pattern of Cp gene expression was reproduced in vitro in the PMC42-LA mammary epithelial cell culture models [146]. The activity level of the Cp gene expression in the cells of the mammary gland does not depend on the availability of copper in the female’s diet or the Cp levels in the blood [147,148,149]. Thus, the cells of the mammary gland produce a tissue-specific molecular form of soluble Cp, and its concentration is strictly regulated at the transcription and splice levels during pregnancy and lactation.

## 7. Milk Ceruloplasmin is a Source of Copper, Which Adapts to ETCM of Newborns

In milk, copper is present in a nondialyzable fraction, and approximately 75–80% of copper is found in Cp [149]. In the colostrum, the Cp molecule binds more labile copper atoms, which can be removed by the copper-specific resin Chelex-100, than in the mature milk. The concentrations of Cp and copper in the milk decrease proportionally during lactation [149,150]. In the human colostrum, Cp and copper concentrations are 150 ± 30 mg/L and 600 ± 200 µg/L, respectively, and they drop to 40 ± 20 mg/L and 150 ± 20 µg/L, respectively, in the transitional milk and decrease by up to ~10% of the initial values in the mature milk. The decrease in the copper concentration follows the increase in the consumed milk volume, so the total quantity of copper in the daily diet of a newborn remains practically invariable. Up to the age of 6 months, an infant typically consumes approximately 1 L of milk, and the concentration of copper is 10 times lower than in the colostrum. We measured milk Cp and copper levels in more than 200 women during the first 10 days of lactation, and only in one case did their concentrations fail to decrease on the 10th day of lactation [150]. In this patient, an A→C point mutation was found in the promoter region of the *Cp* gene (at position—1966). This nucleotide is part of the *cis*-element for transcription factor C/EBPβ (CCAAT/enhancer-binding protein beta), which may potentially take part in the gradual suppression of the Cp gene activity [150]. In Turkish women, the copper concentration in the breast milk decreased in the same manner [151]. In all mammalian species (rats, pigs, dogs, mares, cats, and humans), in which milk copper status indexes were assessed, the Cp and copper concentrations decrease during lactation, and this process does not depend on the copper concentration in the blood [143,149,150,152,153,154,155]. As a result, in breastfeeding, the copper content in a newborn’s food can be approximately maintained at a constant level. Therefore, the decrease of Cp and the Cp-associated copper concentration in milk is a trait that is conserved between and within species. It appears that the decrease in the activity of the *Cp* gene in breast cells during lactation has been preserved by natural selection.

**Figure 1:**
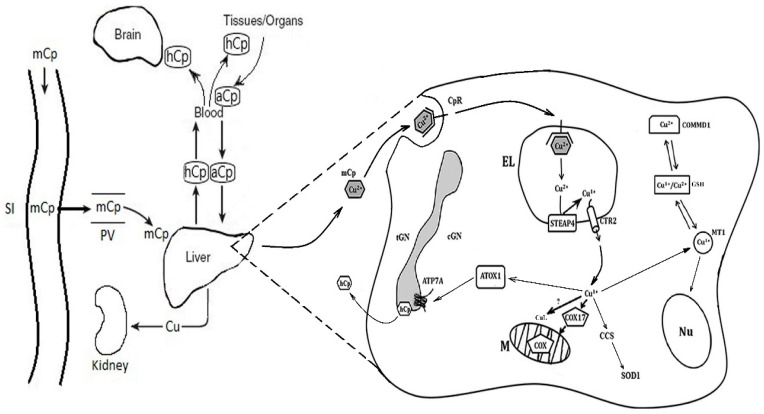
Cartoon scheme of copper turnover in the hepatocytes of newborns. In newborns, milk Cp enters into the gastrointestinal tract and, due to transcytosis, transfers into the bloodstream without modification. Then, it binds with the hepatic Cp receptor and proceeds into the endolysosomes. At pH > 5, the Cu(II) ions are dissociated from the milk Cp molecule, and Cu(II) is reduced to Cu(I) by STEAP4 and imported by CTR2 into the cytosol. Here, Cu(I) is redistributed between Cu(I)-chaperones to be delivered to COX, SOD1, and Cp. In the cytosol, copper is bound by MT1, is involved with the redox cycle MT/GSH, and can be deposited in the hepatocytes. CuL binds the Cu(I) ions, and they are transported into the mitochondria to support copper balance in cytosol. Because MT is found in the mitochondria and nucleus, it is possible that it transfers copper into the nucleus and the mitochondria. Abbreviations on the schema: mCp—milk ceruloplasmin; SI—small intestine; PV—portal vein; hCp—holo-ceruloplasmin; aCp—desialic ceruloplasmin; CpR—ceruloplasmin receptor; Nu—nucleus; M—mitochondria; EL—endolysosome; cGN—cis-Golgi; tGN—trans-Golgi; GSH—glutathione; MT1—metallothionein 1; ATP7A—Cu(I)/Cu(II)-transporting ATPase; CTR2—low affinity copper transporter 2; ATOX1, CCS, and COX17—cytosolic Cu(I)-chaperons for ATP7A, superoxide dismutase 1 (SOD1), and cytochrome-c-oxidase (COX) respectively; STEAP4—six-transmembrane epithelial antigen of the prostate; CuL—low molecular weight carrier of copper; and Cu—copper ions.

The high biological importance of milk Cp is demonstrated by the following facts. Copper atoms that are associated with Cp molecules are assimilated by newborns more easily [143]. The knockout of the *Cp^−/−^* gene in mice causes a decrease in the copper concentration in the milk of females and copper misbalance in the pups [156]. The same effect is produced by mutations of the *Atp7b* gene in the *toxic milk* line of mice [157]. The progeny of *tx*/*tx* mice dies because of the copper deficiency in the mother’s milk, but it completely survives if fed from the first day of life by a wild-type ‘nurse’ [158]. In the gastrointestinal tract of newborns, the milk Cp molecule is not degraded and retains oxidase activity [149], perhaps because in the stomachs of newborns, the pH levels are close to neutral. From the gastrointestinal tract to the bloodstream, milk Cp is transferred without any modifications by transcytosis [149]. A specific endocytic Cp receptor takes part in this process. The same receptor facilitates the capture of milk Cp by the membranes of hepatocytes. The Cp receptor is synthesized in the cells of the small intestine and the liver only at the ETCM stage [159,160]. It is possible that copper is released from milk Cp in the acidic medium of the endolysosomes, and then, it is reduced to Cu(I) by STEAP4 and transported to the cytosol by CTR2. Thus, the copper transport system of newborns is adapted to milk Cp as the source of copper nutrients (Figure 1).

It is worth nothing that the data discussed in this section were obtained from rats. Although the infant liver is different from the rat pup liver with respect to some species-specific features, the facts obtained for rodent copper metabolism are valuable for humans. In both species, at the ETCM stage, the copper status (low copper and ceruloplasmin concentrations in the serum), the copper accumulation in the liver tissue, the excretion of copper with urine but not with feces, and the switching to the ATCM (increased copper and Cp concentrations in the serum, a drop in the liver copper concentration, and the excretion of copper through the bile), as well as the dynamics of the decrease in the copper and Cp concentrations in milk during lactation, are similar. These data allow us to determine that during development, the expression of the copper metabolism genes in the liver and mammary gland develop similarly in rats and humans.

## 8. Specific Features of Copper Metabolism in Newborns Fed Infant Formulas

Children exclusively fed cow milk, which contains copper and milk Cp but at concentrations that are lower than those of human milk, at an age of 6 months develop severe anemia, neutropenia, hypocupremia and display malformation of their bones and defects in erythrocyte maturation and so on [23]. These symptoms can be avoided by adding copper salts to the dry cow milk. Currently, adapted infant formula contains approximately 600 µg of copper per 1 L of the consumed liquid, and that amount roughly corresponds to the concentration of copper in the colostrum. Copper ions are added as inorganic salts, as simple coordination compounds, or as a His_2_Cu(II) complex. In infant formula, the copper is dialyzable, it is not “packed” into Cp, and its concentration in the diet from birth up to 6 months of age does not change. Therefore, newborns fed with such formulas receive copper in a highly mobile form, which easily gets to the bloodstream but is not delivered to the endosomes of hepatocytes. Additionally, the daily copper supply progressively increases along with the volume of consumed food. By the end of the first month, the copper consumption of formula-fed infants exceeds the normal copper consumption of breastfed infants by many times [149]. In rats, which were fed analogous milk formula during the first 1–9 days of life, the transition from ETCM to ATCM occurred earlier than in nursed rats (Table 4, borrowed from our article [149]).

The transition manifests as an abrupt drop in the copper concentration in the liver, an increase in the blood serum copper levels, and preterm activation of the *Cp* gene at the transcription and translation levels. The copper status was also affected in the cerebrospinal fluid. The concentration of copper and Cp was increased by a factor of 7. The specific content of copper in the brain cells did not change. In newborn rats with ETCM and ATCM, which were supplemented with copper ions, the Ctr1, Atp7b, and metallothionein mRNA levels in the liver increased [161]. Simultaneously, the alanine aminotransferase levels elevated, suggesting a risk of copper toxicity with supplementation during infancy. Both experiments with baby formula [149] and copper oral supplementation [161] showed that suckling rat pups were able to adapt to higher amounts of nutritional copper due to changes in the expression of the copper transporters. However, the data also demonstrated that a high concentration of ‘free’ copper ions in the infant food strongly disturbed the copper balance in the cerebrospinal liquid, and brain copper dyshomeostasis is one of the traits of neurodegenerative diseases [162].

Milk proteins are divided into two groups: nutritive proteins, which are used for growth, and bioactive proteins, which perform regulatory and transport functions [163]. In milk, the contents of both are controlled at the level of gene expression in the mammary gland. The concentration of bioactive milk proteins decreases sharply in the first days of lactation [164]. Cp is not listed as a bioactive milk protein [163]. However, the Cp turnover in the newborn body is similar to that of lactoferrin (Lf), the main iron-containing protein of whey milk, which is a bioactive protein [163]. In milk, the Lf concentration falls in the early stages of lactation. The Lf receptor (LfR) gene is expressed in the intestine and in the liver of a newborn only during breastfeeding. This suggests that LfR plays a key role in internalizing Lf and facilitating the absorption of the iron bound to Lf. Polypeptides of Lf are found in the feces of newborns [163,165]. It seems that iron and copper levels, essential and toxic trace elements, are similarly metabolized.

## 9. Conclusions

The reviewed data suggest that the copper imbalance in the early postnatal period, which is induced by feeding infants formula, influences various aspects of copper metabolism. This is primarily an increase in the Cp and copper concentrations in the blood serum and cerebrospinal fluid, but there is no evidence that it can affect the formation of cuproenzymes. It is possible that, in individuals that carry no latent inherited defects in copper homeostasis, the nutrient copper excess is compensated in further development and has no significant impact on health. However, the individuals carrying heterozygous mutations in genes related to copper homeostasis (e.g., heterozygous carriers of Wilson’s disease) may be especially sensitive to copper imbalance in early childhood [166]. The disturbance of regulatory pool copper can influence the signaling, activity of the transcription factors (HIF1, p53, and nuclear hormone receptors), and [Fe–S]-dependent enzymes. Such effects can be very significant, but now their identification is difficult. Because some participants of the CTS are still unknown while some copper-binding proteins are ‘moonlighting’ and their activities depend on the copper level (e.g., Cp, CTR1, ATOX1, and COMMD1) [36,167,168,169,170,171], we may suggest that the differences between breastmilk and infant formula with respect to copper concentrations and copper ‘packaging’ by the Cp protein may be one of the factors that contribute to the negative effects of bottle feeding on the cognitive abilities of children [172,173].

The delayed effects of the impairments of copper homeostasis in early infancy remain poorly studied. If this problem is ignored, there is a risk that it will impact the development of intellectual abilities and physical and mental health. Although many aspects of copper metabolism need further thorough investigation, it may be stated that for the ideal development of the intellectual and physical qualities of the individual, significant attention should be given to the balanced content of copper in the diet of infants.

## Figures and Tables

**Table 1:** The main mammalian cuproenzymes.

Cuproenzyme	Localization (The Main Place)	The Main Functions
SOD1 (Cu(II)/Zn(II)-superoxide dismutase)	Cytosol, nuclear matrix, lysosomes, peroxisomes, mitochondria	Disproportionation of superoxide anions to oxygen and hydrogen peroxide [29]
SOD3 (Cu(II)/Zn(II)-superoxide dismutase)	Extracellular liquids (blood serum, lymph, sclera, etc.)	Antioxidant functions, signaling, stimulation of cell proliferation, decrease of apoptosis, and inflammation [29]
COX (cytochrome-c-oxidase)	Mitochondrial inner membrane	Transfer of electrons from the respiration chain to molecular oxygen [30]
Protein-lysine 6-oxidase (lysyl oxidase)	Extracellular matrix	Oxidation of lysine residues to aldehydes in collagen and elastin precursors [31]
PAM (peptidylglycine α-hydroxylating monooxygenase)	Vesicles, membrane-bound, and soluble forms	Pro-neuropeptide processing by converting to the corresponding amide [32]
DBH (dopamine-β-hydroxylase)	Vesicles, membrane-bound, and soluble forms	Conversion of dihydroxyphenylalanine (DOPA) to noradrenaline [33,34]
Tyrosinase (phenol oxidase)	Melanosomes	Synthesis of melanin from tyrosine [35]
Soluble ceruloplasmin (Cp)	Blood serum, milk, cerebrospinal fluid, and other extracellular liquids	Oxidation of Fe(II) to Fe(III), oxidation of aromatic amines, copper transporter [36,37]
GPI-Cp ^1^ (splice isoform Cp)	Plasma membrane, brain	Oxidation of Fe(II) to Fe(III) [38]
Hephaestin	Plasma membrane, enterocytes	Oxidation of Fe(II) to Fe(III) [39]
Zyklopen	Plasma membranes, placenta	Oxidation of Fe(II) to Fe(III) [40]

^1^ glycosylphosphatidylinositol anchor.

**Table 2:** Mammalian proteins and substances for controlling copper homeodynamics or participating in copper-dependent signaling.

Protein/Substance	Localization	Function
Metallothionein (a large set of isoforms)	Cytosol, mitochondrial matrix, nucleoplasm, blood serum	Detoxification of heavy metals, maintenance of copper and zinc balance, control of apoptosis, and cell protection from death and neoplasia [56,57]
COMMD1 (Copper Metabolism gene MURR Domain 1; previously named MURR1)	Cytosol, nucleoplasm	Excretion of Cu(II) through bile, stabilization of the ATP7B ^1^ structure, and participation in copper-dependent signaling with NF-kB ^2^ [58]
XIAP (X-linked inhibitor of apoptosis protein)	Cytosol	The inhibitor of caspase 3, ubiquitin-ligase related to COMMD1, and a copper level regulator in the cell [45,46]
SCO1/SCO2 (assembly of cytochrome c oxidase 1/2)	Inner mitochondrial membrane	Incorporation of copper ions into COX ^3^, assembly of the COX complex, and control of copper balance in the cells [59,60]
SCC (small copper carrier)	Circulation, urine	Removal of copper from the liver into the bloodstream [61]
CuL (Copper ligand)	Cytosol, mitochondrial matrix	Transfer of copper between the mitochondrial matrix, mitochondrial intermembrane space, and cytosol [62,63]
LOXL (1-4) (lysyl-oxidase-like proteins) transcription factors	Cytosol, nucleus	Copper-dependent suppressors and activators of tumor growth and metastasis [64,65]
Sp1 (specificity protein 1)	Cytosol, nucleus	Multifunctional transcription factor; regulator of CTR1 gene activity [66,67]
MAC1 * (Copper-sensing transcription factor)	Cytosol, nucleus	Transcription factor involved in the regulation of the CTR1 gene [68]
ACE1 * (transcription factor)	Cytosol, nucleus	Regulation of the expression of the metallothionein gene [68]

^1^ copper transporting ATPase, ^2^ nuclear factor kappa-light-chain-enhancer of activated B cells, ^3^ cytochrome c oxidase, * not found in mammals.

**Table 3:** Mammalian copper transporting proteins involved in intracellular copper routes.

Protein	Localization	Function
CTR1 (high affinity copper importer 1)	Plasma membrane homotrimeric integral protein, universal copper importer	Transfer of Cu(I) from the extracellular space to the cytosol (ATP-independent) [72]; Cu^1+^/K^1+^-exchanger; control of morphogenesis [72]; stabilization of the CTR2 structure [73,74,75]
CCS (Cu(I)-chaperon for SOD1)	Cytosol	Transportation of Cu(I) from CTR1 to apo-SOD1 [76]
COX17 (Cu(I)-chaperon for COX)	Mitochondrial intermembrane space	Transportation of Cu(I) from CTR1 to SCO1/SCO2 [77]
SCO1/SCO2	Inner mitochondrial membrane	Insertion of copper to COX; control of copper balance in the cell, implementation of the Cu(II)→Cu(I) redox cycle [59,60]
ATOX1 (Antioxidant 1, Cu(I)-chaperon for ATP7A/B)	Cytosol	Transportation of Cu(I) from CTR1 to the copper-binding motifs of the ATP7A/B [78]; a component of the cytosolic antioxidant system [79], transcription factor [80]
Menkes ATPase (ATP7A, Cu(I)/Cu(II)-transporting ATPase P1 type)	Membranes of the trans-network Golgi complex (except for hepatocytes of adult mammals)	Acceptance of Cu(I) from ATOX1 and its ATP-dependent transfer to the lumen of the Golgi complex; oxidation of Cu(I) to Cu(II) and insertion of copper into extracellular cuproenzymes [81]
Wilson ATPase (ATP7B, Cu(I)/Cu(II)-transporting ATPase P1 type)	Membranes of the trans-network Golgi complex and plasma membrane of the liver, mammary gland, and brain cells	Acceptance of Cu(I) from ATOX1 and its ATP-dependent transfer to the lumen of the Golgi complex; oxidation of Cu(I) to Cu(II) and insertion of copper into ceruloplasmin, copper excretion through bile [81]
CTR2 (low affinity copper transporter 2)	Membranes of the endolysosomes, plasma membrane	Transfer of Cu(I) from lysosomes to the cytosol; regulation of copper import [74,75]
DMT1 (divalent metal transporter 1)	Apical domain of the plasma membrane of the enterocytes and other cells	Transfer of Cu(II)/Cu(I) from GIT ^1^ into enterocytes [82,83]; compensation of CTR1 deficiency [84]

^1^ gastrointestinal tract.

**Table 4:** The effect of baby formula feeding on the copper metabolism of 9-day-old rats.

Characteristics	Control	Experiment
Body weight, g	11.8 ± 1.3 (11)	9.4 ± 1.2 (11)
Serum Cp oxidase activity, mg/100 mL	14.1 ± 2.13 (6)	38.47 ± 4.46 (6) ^§^
Serum Cp antigen activity, mg/100 mL	14.3 ± 1.2 (6)	43.2 ± 2.1 (7) ^§^
Serum Cu concentration, μg/L	300 ± 50 (6)	940 ± 71 (7)
Cu atoms per 1 serum Cp molecule *	4.5	4.5
Cu content in liver, μg/g dry weight	4.58 ± 0.04 (5)	1.91 ± 1.20 (5) ^§^
Cu content in brain, μg	1.71 ± 0.40 (5)	1.72 ± 0.61 (5)
^1^ CSF Cp antigen activity, mg/100 mL ^&^	0.3	2.3
Cu concentration in CSF, μg/L	15	85
Liver total RNA, mg/g tissue	1.7	1.8
Cp-mRNA, μg/mg of total RNA	0.11	0.23
Carbonyl group concentration, A_370_/mg protein: Cytoplasm ^Ψ^ of brain	0.131 ± 0.02 (5)	0.101 ± 0.013 (5)
Cytoplasm of liver	0.093 ± 0.01	0.086 ± 0.07
Serum	0.155 ± 0.01	0.127 ± 0.014

Notes: In parentheses: number of animals; * calculated to immunoreactive Cp; ^&^ measurement made in mixtures from 3 µL aliquots of cerebrospinal fluid from rats of a group, simple mean from three measurements; ^Ψ^ supernatant obtained after sedimentation of tissue homogenate at 23,000× *g* 30 min; ^1^—cerebrospinal fluid, ^§^ *p* < 0.05.
